# Increased Intestinal Permeability and Decreased Resiliency of the Intestinal Barrier in Alcoholic Liver Disease

**DOI:** 10.14309/ctg.0000000000000689

**Published:** 2024-04-25

**Authors:** Garth R. Swanson, Kanika Garg, Maliha Shaikh, Ali Keshavarzian

**Affiliations:** 1 Department of Internal Medicine, Division of Gastroenterology and Hepatology, Medical University of South Carolina, Charleston, South Carolina, USA;; 2 Rush Center for Integrated Microbiome and Chronobiology, Rush University Medical Center, Chicago, Illinois, USA;; 3 Department of Anatomy and Cell Biology, Rush University Medical Center, Chicago, Illinois, USA;; 4 Department of Internal Medicine, Division of Digestive Diseases and Nutrition, Rush University Medical Center, Chicago, Illinois, USA;; 5 Department of Physiology, Rush University Medical Center, Chicago, Illinois, USA.

**Keywords:** intestinal permeability, gut leak, alcoholic liver disease

## Abstract

**INTRODUCTION:**

Only 20%–30% of individuals with alcohol use disorder (AUD) develop alcoholic liver disease (ALD). While the development of gut-derived endotoxemia is understood to be a required cofactor, increased intestinal permeability in ALD is not completely understood.

**METHODS:**

We recruited 178 subjects—58 healthy controls (HCs), 32 with ALD, 53 with AUD but no liver disease (ALC), and 35 with metabolic dysfunction–associated steatotic liver disease (MASLD). Intestinal permeability was assessed by a sugar cocktail as a percentage of oral dose. The permeability test was repeated after an aspirin challenge in a subset.

**RESULTS:**

Five-hour urinary lactulose/mannitol ratio (primarily representing small intestinal permeability) was not statistically different in HC, ALC, ALD, and MASLD groups (*P* = 0.40). Twenty-four–hour urinary sucralose (representing whole gut permeability) was increased in ALD (*F* = 5.3, *P* < 0.01) and distinguished ALD from ALC; 24-hour sucralose/lactulose ratio (primarily representing colon permeability) separated the ALD group (*F* = 10.2, *P* < 0.01) from the MASLD group. After aspirin challenge, intestinal permeability increased in all groups and ALD had the largest increase.

**DISCUSSION:**

In a group of patients, we confirmed that (i) the ALD group has increased intestinal permeability compared with the HC, ALC, or MASLD group. In addition, because small bowel permeability (lactulose/mannitol ratio) is normal, the disruption of intestinal barrier seems to be primarily in the large intestine; (ii) decreased resiliency of intestinal barrier to injurious agents (such as NSAID) might be the mechanism for gut leak in subset of AUD who develop ALD.

## INTRODUCTION

Alcohol has been the most frequently abused drug in the world for centuries (1). According to the 2019 National Survey on Drug Use and Health, an estimated 15 million people in the United States meet criteria for alcohol use disorder (AUD), and the risk of having AUD has been increasing significantly for the past 10 years. Alcohol is considered the third most preventable cause of any disease, and an estimated 95,000 people die from alcohol-related causes annually in the United States (2). The health burden of alcohol is also accompanied by significant economic costs secondary to several factors including health care expenditures and loss of productivity (3,4). Thus, better understanding how unhealthy alcohol consumption results in increased morbidity and mortality is important because it provides an opportunity to identify target(s) for the prevention and/or treatment of alcohol-associated pathology.

One of the primary reasons why unhealthy alcohol consumption and AUD leads to increased morbidity and mortality is that patients with AUD are at an increased risk of organ damage, particularly cirrhosis-related morbidity and mortality (5,6). Alcoholic liver disease (ALD) is a term used to comprise a clinical-histologic spectrum including fatty liver, alcoholic hepatitis, and cirrhosis with its complications (7). ALD is one of the main causes of chronic liver disease worldwide and accounts for up to 48% of cirrhosis-associated deaths in the United States (8). Clinically significant ALD develops in approximately 20%–30% of, but not all, those with AUD (9,10).

While the likelihood of developing ALD increases with increased alcohol consumption, extensive individual variability exists (7) indicating that while excessive alcohol consumption is necessary for the development of ALD, it alone is not sufficient in inducing ALD. Several experiment studies in animal models and human (11,12) have demonstrated that inflammation is required for alcohol-induced liver cell injury and clinically relevant ALD. One of the primary sources of systemic and hepatic inflammation in AUD seems to be alcohol-induced gut microbiota dysbiosis and disruption of intestinal barrier (gut leakiness to endotoxins and other proinflammatory products of gut microbiota) (13,14). Indeed, studies in humans have demonstrated that heavy and unhealthy alcohol use leads to increased intestinal permeability (15–19). We were the first to show in 1999 that only a subset of patients with AUD who also had liver disease had increased intestinal permeability (16). We (16) and others (17) have suggested that this increase in intestinal permeability enhances the translocation of endotoxins and bacterial products, which contributes to the inflammatory process required to develop ALD. Several animal and human studies have highlighted the key role alcohol-induced intestinal hyperpermeability plays in the development of ALD (15–17,20). Increased alcohol-induced intestinal permeability leads to an increase in gut-derived bacterial endotoxins in the blood, bacterial translocation, inflammation, and liver injury (21). The development of this gut-derived endotoxemia (and other proinflammatory products of gut microbiota) is a pivotal required cofactor for ALD (22–24).

However, the reason why only a subset of patients with AUD develops disruption of the intestinal barrier's integrity and organ damage such as ALD is not fully understood. We posit that: (i) alcohol-induced disruption of intestinal barrier integrity is primarily in the colon because we and others have shown that gut microbiota dysbiosis could be one of the mechanisms of gut leak (13–17,20,25–28), and microbiota primarily reside in the colon, and (ii) disruption of the intestinal barrier's integrity by unhealthy alcohol consumption might not be enough to cause sustained and clinically relevant intestinal leak to bacterial products; but unhealthy alcohol consumption could decrease the resiliency of the intestinal barrier to other injurious factors such as Non-steroidal anti-inflammatory drugs (NSAIDs) (commonly used by patients with AUD to treat hangovers) in a subset of patients with AUD who will be at risk of developing organ damage such as ALD. We conducted a cross-sectional, prospective, and observational study. Accordingly, the aim of our study was to test our hypothesis by assessing intestinal permeability before and after an aspirin challenge in patients with AUD and no liver disease (ALC), patients with AUD and liver disease ALD, and patients with metabolic dysfunction–associated steatotic liver disease (MASLD) to: (i) identify the primary site of the leak in patients with AUD, (ii) evaluate whether patients with ALD have increased intestinal permeability compared with those with MASLD, (iii) establish the most robust urinary sugar marker(s) that could differentiate ALD from ALC and (iv) determine whether those with ALD have decreased resiliency of their intestinal barrier and are thus at risk of liver injury with unhealthy alcohol intake.

## METHODS

### Study subjects

The study was registered on Clinicaltrials.gov #NCT05428072, approved by the Rush University Institutional Review Board #09042105. All the participants signed an informed consent form. This study investigated a total of 178 adult (older than 21 years) subjects. Of them, 58 subjects were healthy controls (HCs). Fifty-three of the subjects were individuals with AUD without liver disease ALC, 32 were individuals with AUD with liver disease ALD, and 35 were individuals with MASLD. Subjects with AUD were recruited from the detox unit and halfway house near Rush University Medical Center (RUMC) and those with ALD from Gastroenterology and Hepatology outpatient clinics at RUMC. Healthy subjects were recruited from homeless shelters in the Chicagoland area, Craig's List, and from Gastroenterology and Hepatology clinics at RUMC.

All subjects completed a baseline demographic form that included age, gender, body mass index race, years of education (as an indirect measure of socioeconomic status), smoking history, and self-reported use of illicit drugs. All subjects also completed the Lifetime Drinking History (LDH) assessment (29,30). All subjects had blood drawn during recruitment to assess biochemical markers of liver injury including serum aspartate amino transferase, amino alanine transferase, alkaline phosphatase, and total bilirubin. All questionnaire packets were labeled by a sequential patient number to maintain confidentiality and served as the patient identifier for the remainder of the study.

### Inclusion criteria

A history of alcohol consumption was assessed by a validated National Institute on Alcohol Abuse and Alcoholism (NIAAA)-endorsed assessment instrument—LDH (29,31). Subjects with ALC and ALD fulfilled AUD as defined by the LDH assessment (craving, loss of control, or negative emotional state related to alcohol consumption) and regular and heavy alcohol use for at least 3 months during the 6 months before enrollment. Based on self-reports, all AUD subjects were adhering to complete alcohol abstinence for at least 7 days before the permeability tests. We required that patients remained abstinent from alcohol for at least 7 days before permeability testing to prevent the acute effects of alcohol consumption to confound our ability to determine a sustained gut leak, i.e., the presence of disrupted intestinal permeability even after 7 days of not drinking alcohol. We also chose 7 days of abstinence to avoid the potential impact of withdrawal symptoms and associated stress on the intestinal barrier. Sobriety was confirmed by the LDH assessment and by a negative blood alcohol level. Liver disease in ALD and MASLD was defined as the presence of elevated amino alanine transferase or aspartate amino transferase levels that were >1.5 times normal or clinical or radiological (computed tomography or ultrasound) evidence of liver disease. Although liver histology is optimal for establishing the presence of liver disease and the degree of liver injury and fibrosis, for ethical reasons, liver biopsy could not be performed in subjects without biochemical or clinical evidence of liver disease or in those with AUD with evidence of liver disease when histological diagnosis was not clinically indicated. HCs were otherwise healthy individuals with no known liver disease, had no history of unhealthy alcohol consumption and who did not fulfill any of the exclusion criteria, and who were willing to participate in the study.

### Exclusion criteria

Subjects were excluded for the following criteria: (i) unreliable drinking history; (ii) significant renal impairment (creatinine > l.2 mg/dL); (iii) GI disease or other diseases that affect GI motility such as scleroderma; (iv) diabetes; (v) clinically detectable ascites that can affect renal function and volume distribution of sugar markers; (vi) significant peripheral edema that can affect renal function and volume distribution of sugar markers; (vii) sepsis; (viii) clinically significant cardiac failure (New York classification stage III/IV); (ix) regular daily use of medications that may affect intestinal permeability such as NSAIDs or intestinal motility (e.g., metoclopramide); (x) ALD positive for other markers of liver disease such as smooth muscle antibody, hepatitis B surface antigen, or hepatitis C antibody; and (xi) patients with low platelet count (<80k), uncorrectable prolonged prothrombin time (PT) (>15 seconds), and history of bleeding were excluded from aspirin tests. None of the subjects were taking lactulose or other sugar substitutes regularly.

### Measures

#### Lifetime drinking history.

The LDH is a structured interview that is designed to provide quantitative indexes of an individual's alcohol consumption patterns from the onset of regular drinking. Attention is focused on quantity, frequency, variability in consumption, types of beverages, and life events that mark a change in drinking pattern. Solitary vs social drinking and time of day when alcohol is consumed are also recorded. The interview takes 20–30 minutes to complete and has been shown to be a validated measure of alcohol consumption (32).

#### Intestinal permeability.

Intestinal permeability was assessed as previously described (33). In brief, all subjects were asked to avoid consuming lactulose, sugar substitutes, diet foods, and foods that might contain mannitol or sucralose for 72 hours before the study visit. Subjects then fasted overnight. In the morning, the subjects orally ingested a test solution. The solution was a cocktail of 2 g of mannitol, 7.5 g of lactulose, and 2 g of sucralose in 300 mL of water. The subjects fasted for 2 hours after the start of urine collection and then were asked to eat normally except for refraining from lactulose, sugar substitutes, and sucralose or mannitol–containing products, during the 24-hour urine collection period. Subjects were asked to empty their bladder before consuming the test solution. After consuming the solution, subjects collected their urine for 24 hours. Two urine collections were recorded: 0–5 hours and 0–24 hours. Urine volumes were recorded and stored until analysis. Five-hour urinary lactulose, mannitol, and lactulose-to-mannitol (L/M) ratio are primarily markers of small bowel permeability; and 24-hour urinary sucralose and lactulose excretion are markers of total gut permeability, with sucralose primarily representing colonic permeability (34). This is because of both sucralose and lactulose being able to permeate through both the small and large intestines (colon). However, sucralose is not fermented by colonic bacteria, whereas 75% of lactulose and mannitol are fermented by colonic bacteria (35).

To examine the intestinal permeability immediately after exposure to an injurious agent (aspirin), we did a second assessment of intestinal permeability after an aspirin challenge, as we have previously published (18). In brief, 2 weeks after the baseline collection, subjects were asked to do a second collection and were given the sugar cocktail along with 4 tablets of aspirin each containing 325 mg for the aspirin challenge. Urine samples were analyzed by gas chromatography as previously described (33). The intestinal permeability was measured using an Agilent 6890 GC equipped with a flame ionization detector.

### Analytical methods

Urine samples of the subjects were analyzed for sugar content using gas chromatography, following the conversion of sugars and methylated sugars to their alditol acetate derivatives as previously described (33). The fractional excretion of mannitol, lactulose, and sucralose was calculated based on the urinary concentration of these sugars in mg/mL. The percent excretion of oral intake was then calculated for each sugar. *T* tests and Analysis of Variance (ANOVA) tests were used for comparison between the 4 groups. For intestinal permeability measurements, ∼5 percent of patients were excluded from statistical analysis as outliers. Outliers were defined as > 2 SD above the mean. All statistical analyses were conducted in GraphPad Prism (v9.1.1) and SPSS (v26).

## RESULTS

### Clinical assessments

A total of 178 patients completed the study. Fifty-eight subjects were HCs, 53 were individuals with AUD without liver disease (ALC), 32 were individuals with AUD with liver disease ALD, and 35 were individuals with MASLD. Ninety-four subjects were male (53%) and 84 were female (47%). One hundred three were Caucasian (60%), 48 were African American (27%), 11 were Asian (6%), 11 were Hispanic (6%), and 5 did not provide ethnicity (3%). The mean age was 47.4 ± 13.4 years. The mean BMI was 28.1 ± 6.2 kg/m^2^. Table 1 summarizes the demographics and clinical features of all study participants in each group.

**Table 1.** Subject characteristics

	HC N = 58	ALC N = 53	ALD N = 32	MASLD N = 35	*P* value
Age (mean ± SD)	42.0 ± 14	46.7 ± 12.5	52.3 ± 11.6	55.0 ± 9.2	0.00
Gender (male: female)	25:33	35:18	21:11	13:22	0.02
Ethnicity (White: other: not stated)	32:26:0	27:24:2	25:6:1	19:14:2	0.1
BMI (kg/m^2^)	28.0 ± 6.4	27.7 ± 6.8	27.6 ± 6.4	29.4 ± 5.7	0.7

ALC, AUD but no liver disease; ALD, alcoholic liver disease; HC, healthy control; MASLD, metabolic dysfunction–associated steatotic liver disease.

### Five-hour lactulose, mannitol, and lactulose/mannitol ratio (markers of small intestinal permeability)

**Figure 1.**
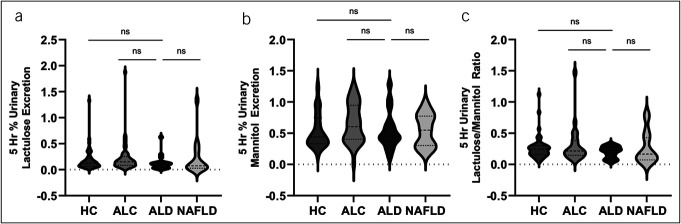
Unhealthy alcohol use did not significantly affect small intestinal permeability. (**a**) The 5-hour urinary lactulose (% excretion of oral dose) per group. There was no statistical difference between groups (*P* value = 0.63). (**b**) The 5-hour urinary mannitol (% excretion of oral dose) per group. There was no statistical difference between groups (*P* value = 0.22). (**c**) The ratio of 5-hour urinary lactulose/5-hour urinary mannitol (% excretion of oral dose) per group. There was no statistical difference between groups (*P* value = 0.40).

Unhealthy alcohol use did not significantly affect small intestinal permeability. The 5-hour urinary lactulose (% excretion of oral dose) was not statistically different between groups (*P* value = 0.63): HC 0.19 ± 0.22, ALC 0.23 ± 0.29, ALD 0.13 ± 0.14, and MASLD 0.22 ± 0.34. The 5-hour urinary mannitol (% excretion of oral dose) was not statistically different between groups (*P* value = 0.22): HC 0.53 ± 0.28, ALC 0.66 ± 0.31, ALD 0.54 ± 0.30, and MASLD 0.54 ± 0.26. The 5-hour L/M ratio in each category was not statistically different between groups (*P* value = 0.40): HC 0.27 ± 0.19, ALC 0.31 ± 0.30, ALD 0.20 ± 0.09, and MASLD 0.27 ± 0.26 (Figure 1). Historically, the L/M ratio has been used to measure small bowel permeability. However, in this study, 5-hour L/M ratio was not found to be a useful measure of distinguishing between ALD and the other groups of patients.

### Twenty-four hour lactulose, sucralose, and sucralose/lactulose percent ratio (markers of total, primarily colonic, gut permeability)

**Figure 2.**
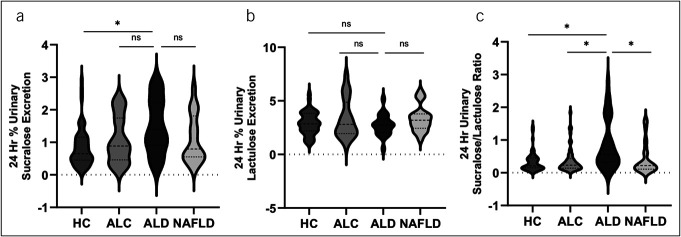
Total gut permeability (primarily colon permeability) was significantly increased in patients with alcohol use disorders. (**a**) The 24-hour urinary lactulose (% excretion of oral dose) per group. There was no statistical difference between groups (*P* value = 0.26). (**b**) The 24-hour urinary sucralose (% excretion of oral dose) per group. There was statistical difference between groups (*F* = 5.3, *P* value < 0.01). (**c**) The ratio of 24-hour urinary sucralose/24-hour urinary lactulose (% excretion of oral dose) per group. There was statistical difference between groups (*F* = 10.2, *P* value < 0.01).

Total gut permeability (primarily colon permeability) was significantly increased in patients with AUDs. The 24-hour urinary lactulose (% excretion of oral dose) was not statistically different between groups (*P* value = 0.26): HC 2.91 ± 1.06, ALC 3.34 ± 1.82, ALD 2.72 ± 0.99, and MASLD 3.33 ± 1.21. However, the 24 hour-urinary sucralose (% excretion of oral dose) was statistically different between groups (*F* = 5.3, *P* value < 0.01): HC 0.85 ± 0.60, ALC 1.09 ± 0.72, ALD 1.48 ± 0.83, and 1.10 ± 0.73. The 24-hour sucralose/lactulose (S/L) ratio was also statistically different between groups (*F* = 10.2, *P* value < 0.01) (Figure 2), and the result in each category was as follows: HC 0.33 ± 0.28, ALC 0.38 ± 0.38, ALD 0.87 ± 0.77, and MASLD 0.42 ± 0.47. These data showed that the 24-hour urinary sucralose was different in the ALD group. Our data also showed that the 24-hour S/L ratio was a more sensitive marker than sucralose to separate ALD from the other 3 patient groups: HC, ALC, and MASLD. We have previously shown that S/L ratio is a better marker of total intestinal permeability (primarily colon permeability) than 24-hour urinary sucralose in patients with Parkinson disease (33). Neither gender nor BMI were significant covariates in the analysis of 24-hour urinary sucralose (*P* value 0.954 and 0.369, respectively).

### Post aspirin challenge

Intestinal barrier resiliency was assessed in 40 of the 178 study cohorts who agreed to take aspirin and have a second permeability test. We did not offer the aspirin permeability test to those patients with liver disease who had either low platelets, high international normalized ratio (INR), history of GI bleed, or varices due to risk of bleeding. Of these 40 patients, 12 were HCs, 7 had ALD, 14 had ALC, and 7 were those with MASLD.

**Figure 3.**
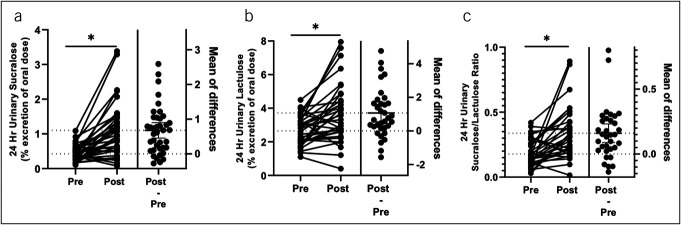
Aspirin challenge caused a marked increase in permeability in all groups. (**a**) The 24-hour urinary lactulose (% excretion of oral dose) pre vs post aspirin (*P* value < 0.01, paired analysis). (**b**) The 24-hour urinary sucralose (% excretion of oral dose) pre vs post aspirin (*P* value < 0.01, paired analysis). (**c**) The 24-hour urinary sucralose/lactulose ratio pre vs post aspirin (*P* value < 0.01, paired analysis).

**Figure 4.**
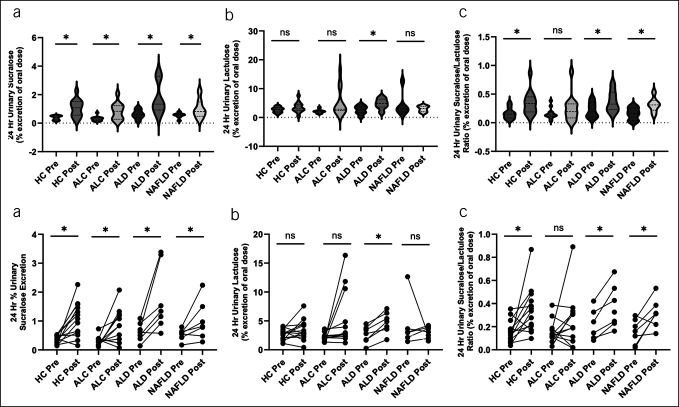
(**a**) Results of the aspirin challenge by group as violin graphs. (**a**) The 24-hour urinary sucralose (% excretion of oral dose) pre vs post aspirin by group. There was a statistically significant increase in all groups (*P* value < 0.05). (**b**) The 24-hour urinary lactulose (% excretion of oral dose) pre vs post aspirin by group. There was a statistically significant increase in the ALC and ALD groups (*P* value < 0.05) but not in the HC or MASLD group (*P* = 0.16 and *P* = 0.84, respectively). (**c**) The 24-hour sucralose/lactulose ratio pre vs post aspirin by group. There was a statistically significant increase in HC, ALD, and MASLD groups (*P* value < 0.05), but not in the ALC group. (**b**) Results of the aspirin challenge by group as line graphs. (**a**) The 24-hours urinary sucralose (% excretion of oral dose) pre vs post aspirin by group. There was a statistically significant increase in all groups (*P* value < 0.05). (**b**) The 24-hour urinary lactulose (% excretion of oral dose) pre vs post aspirin by group. There was a statistically significant increase in ALC and ALD groups (*P* value < 0.05) but not in the HC or MASLD group (*P* = 0.16 and *P* = 0.84, respectively). (**c**) The 24-hour sucralose/lactulose ratio pre vs post aspirin by group. There was a statistically significant increase in HC, ALD, and MASLD groups (*P* value < 0.05), but not in the ALC group. ALC, AUD but no liver disease; ALD, alcoholic liver disease; HC, healthy control; MASLD, metabolic dysfunction–associated steatotic liver disease.

The aspirin challenge caused a marked increase in total permeability in all groups. The 24-hours urinary lactulose (% excretion of oral dose) pre vs post challenge was 2.64 ± 0.85 vs 3.4 ± 1.57 (*P* value < 0.01, paired analysis); the 24-hour urinary sucralose (% excretion of oral dose) pre vs post challenge was 0.42 ± 0.23 vs 1.10 ± 0.76 (*P* value < 0.01, paired analysis); and the 24-hour urinary S/L ratio pre vs post challenge was 0.18 ± 0.10 vs 0.33 ± 0.20 (*P* value < 0.01, paired analysis) (Figure 3). By group (Figure 4A, 4B), the 24-hour urinary sucralose (% excretion of oral dose) pre vs post challenge was significantly increased in all groups (*P* value < 0.05): HC 0.37 ± 0.15 vs 1.05 ± 0.60, ALC 0.31 ± 0.17 vs 0.84 ± 0.58, ALD 0.62 ± 0.32 vs 1.74 ± 1.13, and MASLD 0.43 ± 0.22 vs 1.01 ± 0.67. In addition, by repeated measures ANOVA, ALD was significantly increased from the other groups (*F* = 3.37, *P* value < 0.05). The 24-hour urinary lactulose (% excretion of oral dose) pre vs post challenge was significantly increased in the ALC and ALD groups (*P* value < 0.05) but not in the HC or MASLD group (*P* = 0.16 and *P* = 0.84, respectively): HC 2.75 ± 0.92 vs 3.28 ± 1.79, ALC 2.36 ± 0.73 vs 2.89 ± 1.11, ALD 2.93 ± 0.90 vs 5.03 ± 1.60, and MASLD 2.64 ± 0.84 vs 3.40 ± 1.57, . The 24-hour S/L ratio pre vs post challenge was significantly increased in HC, ALD, and MASLD (*P* value < 0.05), but not ALC: HC 0.16 ± 0.10 vs 0.34 ± 0.20, ALC 0.17 ± 0.11 vs 0.31 ± 0.24, ALD 0.22 ± 0.32 vs 0.38 ± 0.20, and MASLD 0.21 ± 0.07 vs 0.31 ± 0.15. Therefore, under exposure to the injurious agent aspirin, urinary sugar probes were significantly increased in all groups, but aspirin-induced disruption of intestinal permeability was more in patients with ALD.

## DISCUSSION

Based on our literature review of the PubMed database, we identified 6 reports that studied intestinal permeability in patients with liver disease. Sample size in these studies ranged from 12 to 83; however, the largest ALD group was only 20 patients, and patients with ALC were not separated from ALD (36–41). Thus, to the best of our knowledge, our study is the largest cohort to evaluate intestinal permeability in patients with AUD, both ALC and ALD.

In this cohort, we found that: (i) in this cohort of patients with AUD, we confirmed prior studies (16,17,21,42) that ALD is associated with gut leak. The site of this leak in patients with ALD seems to be primarily colon because patients with ALD did not have increased 5-hour urinary mannitol or lactulose or L/M ratio that primarily represent small bowel permeability but had increased 24-hours urinary sucralose that represent total gut permeability (34), (ii) the intestinal barrier is more disrupted in ALD than nonalcoholic liver diseases such as MASLD, which also is confirmed by prior studies (16), (iii) the 24-hour urinary S/L ratio seems to be the most robust combination of urinary sugar markers that could differentiate ALD from ALC and ALD from MASLD. This finding is similar to our prior study where we found that the S/L ratio is a better permeability marker than 24-hour urinary sucralose to distinguish patients with Parkinson disease from age-matched controls (33). This is not surprising because the use of 2 sugar probes with similar kinetics, as in lactulose and sucralose (33), could eliminate other factors that are not relevant to intestinal permeability but still affect urinary sugar levels such as intestinal transit, volume of distribution of sugar markers, renal function. and completeness of urine collection (34,43), and (iv) those with ALD have decreased resiliency of their intestinal barrier (primarily colon) with aspirin challenge. This finding provides a potential mechanism by which patients with AUD might develop gut leak that puts them at risk of developing organ damage such as ALD.

Disrupted gut barrier function and increased intestinal permeability have been associated with several different diseases, including ALD (34,44–46). Multiple studies in human and animal models have interrogated the mechanisms of alcohol-induced disruption of intestinal barrier (16,22,47,48). The intestine is the largest interface between the environment and the body (49), a single layer of intestinal epithelial cells regulated by specialized transmembrane structures called tight junction proteins and adherens junctions that associate the actin cytoskeleton to form multifunctional dynamic complexes called the apical junctional complex. Alcohol can induce oxidative injury to the intestinal epithelial cells and disrupt the apical junctional complex including zonula occluding–1, occludin, claudin, and E cadherins and cytoskeletal protein actin that leads to disruption of the intestinal barrier (47,50), partly a consequence of alcohol-induced nuclear factor-κB (NF-κB) activation and inducible nitric oxide synthase (iNOS) upregulation (48).

Given the prevalence of AUD in United States, we wanted to determine the optimal urinary sugar markers to assess intestinal permeability in patients with AUD and ALD. Sugar probes are used mainly to assess the paracellular pathway (vs transcellular permeability) in the intestine (33,34,51). These sugars have different kinetics, different tertiary molecular structures, and are differently metabolized by bacteria (34). Therefore, somewhat unsurprisingly, despite numerous studies, there is no one universal test considered to be the gold standard for measuring permeability. In addition, for *in vivo* permeability measurements, there are multiple variables that can affect the movement of probes across the paracellular pathway, including the concentration gradient across the barrier, the surface area of the epithelium, the time available for permeation, and the intrinsic permeability properties of the barrier (34). The sugar probes typically used in studying intestinal permeability include monosaccharides (sucrose, mannitol) and disaccharides (lactulose, sucralose). Sucrose is metabolized by intestinal brush border enzymes rapidly after ingestion; thus, it is proposed for measuring gastroduodenal permeability (52). Mannitol is a probe to assess permeability in the small intestine (primarily proximal small intestine), lactulose to assess small intestine, and sucralose, which is not metabolized by the colonic bacteria, to assess total gut permeability (primarily colon) (34,35,53).

Renal function can also affect the urinary excretion of certain sugar probes. In this study, we excluded patients with a creatinine of >1.2, though this does not necessarily exclude those with more subtly impaired renal function. We previously compared urinary sugar values as a percent excretion with values corrected for glomerular filtration rate and found no difference if the creatinine is < 1.2(33). Thus, we do not expect that more subtle renal dysfunction significantly affected our results. Furthermore, impaired renal function lowers urinary sugars and thus our conclusion that patients with ALD have increased intestinal permeability (based on our observed increased urinary sucralose) should not be affected by subtle renal dysfunction. None of our study subjects had low serum creatinine.

For intestinal permeability, the 5-hour L/M ratio is most commonly used in celiac disease (52,54), where there is a decrease in the surface area of the small intestine with ablation of villi. This is because there is decreased mannitol excretion due to decreased surface area and an increase in leak pathway in the crypts with increased lactulose excretion. Thus, the increase in the ratio of L/M represents increased intestinal permeability. Based on this model, the L/M ratio has been shown to be useful to measure intestinal permeability in other diseases of the small bowel, even in models of severe dysbiosis, such as Crohn's disease (55). However, many disorders such as ALD are not associated with villous atrophy/shortened villi and thus marked changes in intestinal surface area. Indeed, alcohol-induced barrier dysfunction is not a disease that is caused solely by destruction of the villi (10), and several studies suggested that barrier disfunction in ALD is associated with disrupted colonic permeability (16,17,21,42). Thus, use of mannitol, lactulose, and L/M ratio does not seem the ideal probe to assess intestinal barrier function in ALD. Rather, probes such as sucralose that are not primarily affected by surface area and can also assess colon permeability (because it does not get metabolized by bacteria) seem to be a more suitable probe for the assessment of intestinal permeability in disorders such as ALD. Indeed, we (56) and others (57) have shown the usefulness of urinary sucralose to assess intestinal permeability in humans. Furthermore, we have used urinary sucralose and demonstrated gut leak in animal model of ALD (58). There is additional rationale for the use of sucralose to assess the intestinal barrier in disorders associated with microbiota dysbiosis such as AUD and ALD. Sucralose, unlike mannitol and lactulose, is not metabolized by gut bacteria, and thus the presence of dysbiosis that could affect urinary mannitol and lactulose independent of changes in intestinal permeability will not affect urinary sucralose. Moreover, in this study, we show the ratio of urinary S/L was better able to differentiate ALD from ALC and MASLD. It is not surprising due to the principle of differential urinary excretion. We previously showed that the kinetics of sucralose and lactulose are similar and that S/L is a better marker for intestinal permeability (33). Our findings therefore support the use of the S/L ratio to assess intestinal permeability in AUDs.

The intestinal barrier is a dynamic barrier that has a highly variable function over different states. While at steady state, certain inert sugar probes may be optimal for the assessment of intestinal permeability, under conditions of acute stress or acute exposure to an injurious or toxic agents such as alcohol or NSAIDs, the optimal assessment of the intestinal barrier may change. Our study showed that aspirin worsens the intestinal barrier integrity in all study subjects, but worsening of barrier function by aspirin was more marked in those with ALD. This suggests that in a subset of unhealthy alcohol consumption, there is decreased resiliency of intestinal barrier making the host more susceptible to increased intestinal permeability, gut-derived endotoxemia, and end-organ damage. This 2-hit hypothesis might explain why unhealthy alcohol consumption increases the risk of more severe liver disease in patients at risk of other forms of liver disease such as human immunodeficiency virus (59), viral hepatitis (60), and metabolic dysfunction–associated steatotic liver disease (61).

Our study did have several important limitations. First, we assessed intestinal barrier function by inert sugar probes only. We did not directly biopsy the colon or small bowel to compare the intestinal barrier apical junction structure or function. Second, urine collections for intestinal permeability measurements were performed at home by the patients and were not directly observed. Therefore, we had 3%–5% of our samples that were either incorrectly collected, mislabeled, or the sugars were not properly taken. The breakdown of which study groups these subjects were in is demonstrated in Supplementary Digital Content (see Supplementary Table 1, http://links.lww.com/CTG/B92). Third, in our comparison with the baseline, we had a much smaller number of subjects complete the post-aspirin challenge (178 vs 40). This was particularly true in the ALD and MASLD groups, where thrombocytopenia and elevated PT limited recruitment. Therefore, our data on the aspirin challenge and intestinal permeability need to be evaluated with this limitation in mind. Fourth, the subjects with AUD in this group were abstinent from alcohol for a variable amount of time (greater than 7 days), but the time from last drink was not standardized. Fifth, not all patients in the ALD group had a biopsy performed, and elastography was also not performed to confirm the degree of fibrosis. Finally, sample size for the individual subgroups were small, and several demographic characteristics were different among subgroups. Future studies with larger subgroup sample sizes with matched controls are required to confirm our findings. In addition, future studies could include multiple measures of permeability to corroborate that differences in sugar probe tests represent barrier function changes.

In summary, the main findings of this study are as follows: (i) whole gut (primarily colonic) permeability is increased in ALD but not in ALC, or MASLD, which further supports that ALD is driven by intestinal barrier dysfunction, bacterial translocation, and endotoxemia; (ii) we found that at steady state, the use of 2 inert sugars with similar kinetics (24 hours S/L ratio) was useful in differentiating patients with AUD with and without ALD; and (iii) after an aspirin challenge, 24-hour urinary sucralose markedly increased in all groups, but particularly in those with ALD, suggesting that unhealthy alcohol consumption decreases the resiliency of the intestinal barrier. Decreased intestinal barrier resiliency to commonly exposed injurious agents (e.g., NSAIDs, stress) could in turn increase the risk of alcohol-induced organ damage such as liver disease.

## CONFLICTS OF INTEREST

**Guarantor of the article:** Ali Keshavarzian, MD.

**Specific author contributions:** A.K.: study concept and design, subject recruitment, subject assessment, and data interpretation. G.S.: acquisition of the data, statistical data analysis, and interpretation of the data. M.S.: acquisition of the data, analysis of permeability data. G.S. and K.G.: drafting of the manuscript. G.S. and A.K.: critical revision of the manuscript. All authors approved the final version of the manuscript.

**Financial support:** This study was supported by the National Institute on Alcohol Abuse and Alcoholism (R01AA013745-01A1, 1RC2AA019405-01, R24AA026801: A.K.). This research was also supported partly by philanthropic funding to A.K. from Mr. and Mrs. Larry Field, Mr. and Mrs. Glass, Mrs. Marcia and Mr. Silas Keehn, the Sklar Family, the Johnson Family, and Mr. Harlan Berk.

**Potential competing interests:** None to report.

**IRB approval:** This study was approved by the Rush University Institutional Review Board (IRB) #09042105.

**Study Highlights**

**WHAT IS KNOWN**

✓ Alcohol use increases intestinal permeability.
✓ Increased intestinal permeability is linked to multiple diseases.

**WHAT IS NEW HERE**

✓ Gut leak is mainly in the large intestine in those with alcoholic liver disease.
✓ Sucralose/lactulose ratio was the optimal marker to distinguish alcoholic liver disease from alcohol use disorder without liver disease, metabolic dysfunction–associated steatotic liver disease, and healthy controls.
✓ Those with alcoholic liver disease have decreased resiliency to injurious agents.

## Supplementary Material
