# Supplementary material for: Increased Intestinal Permeability and Decreased Resiliency of the Intestinal Barrier in Alcoholic Liver Disease
Source: Clin Transl Gastroenterol. 2024 Apr 25;15(4):e00689. doi: 10.14309/ctg.0000000000000689 (PMC11042778; doi:10.14309/ctg.0000000000000689)
Supplement: Supplementary file 1 [file ct9-15-e00689-s001.docx]

| Supplementary Table 1: Incomplete or No Collection by Group | | | | |
| --- | --- | --- | --- | --- |
|  | **HC** | **ALC** | **ALD** | **NAFLD** |
| Incomplete Collection | **2** | **12** | **5** | **7** |
| No Collection | **3** | **10** | **2** | **1** |
